# Periodontal Molecular Diagnostics: State of Knowledge and Future Prospects for Clinical Application

**DOI:** 10.3390/ijms252312624

**Published:** 2024-11-25

**Authors:** Ewa Dolińska, Patryk Wiśniewski, Małgorzata Pietruska

**Affiliations:** 1 Department of Periodontal and Oral Mucosa Diseases, Medical University of Bialystok, ul. Waszyngtona 13, 15-269 Bialystok, Poland; mpietruska@wp.pl; 2 Student’s Research Group at the Department of Periodontal and Oral Mucosa Diseases, Medical University of Bialystok, ul. Waszyngtona 13, 15-269 Bialystok, Poland; patryk.wisniewski@umb.edu.pl

**Keywords:** periodontitis, gingivitis, gingival crevicular fluid, saliva, biomarkers, point-of-care diagnostics

## Abstract

Periodontitis leads to immunologically mediated loss of periodontium and, if untreated, can result in tooth loss. Periodontal diseases are the most prevalent in the world and have a very strong impact on patients’ well-being and general health. Their treatment generates enormous costs. Given the above, precise, prompt, and predictive diagnosis of periodontal disease is of paramount importance for clinicians. The aim of the study was to summarize the state-of-the-art knowledge of molecular periodontal diagnostics and the utility of its clinical application. There is a great need to have diagnostic tests that not only describe the periodontal destruction that has occurred in the tissues but also allow clinicians to detect disease at a subclinical level before the changes occur. A test that would enable clinicians to follow the course of the disease and detect areas prone to exacerbation could be used to evaluate the effectiveness of ongoing periodontal therapies. Unfortunately, there is no such diagnostic method yet. A hopeful prospect is molecular diagnostics. There are numerous studies on biomarkers of periodontal disease. Point-of-care tests are also emerging. There are possibilities for processing large biological datasets (omics data). However, all of the above have a minor role in the overall single-patient diagnostics process. Despite advances in microbiological, molecular, and genetic research, the basis of periodontal diagnosis is still clinical examination enriched by the evaluation of radiological images.

## 1. Introduction

**Figure 1:**
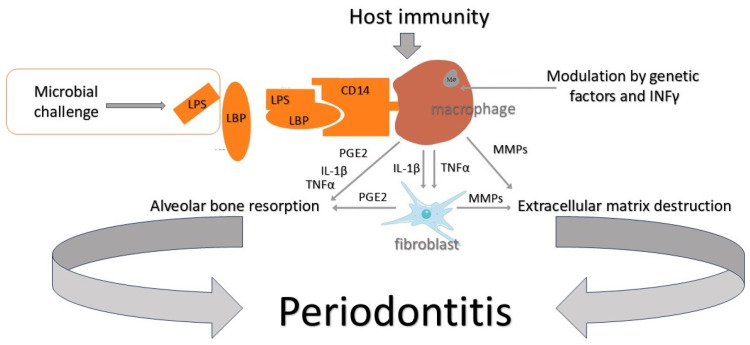
Schematic illustration of the destruction of the alveolar bone and connective tissue in the course of periodontitis. Lipopolysaccharide (LPS), a major virulence factor for Gram-negative bacteria, binds to the lipid-binding protein (LBP). This complex is recognized by the CD14 receptor on monocytes/macrophages. Association of the LPS–LBP complex with the CD14 receptor initiates trans-membrane signaling that activates the cell to synthesize and secrete prostaglandin 2 (PGE2), the cytokines TNFα and IL-1β, and extracellular matrix metalloproteinases (MMPs). TNFα and IL-1β bind to receptors on fibroblasts and initiate signaling for the synthesis and secretion of MMPs and PGE2. MMPs mediate the loss of gingival extracellular matrix and periodontal ligament, while PGE2 leads to bone destruction. TNFα and IL-1β can also directly influence minor bone loss.

Inflammatory periodontal diseases, among them gingivitis and periodontitis, are the most prevalent in the world [1]. It is estimated that these diseases affect 743 million people worldwide and their advanced forms affect 11% of the population [2]. Periodontal disease leads to immunologically mediated loss of soft and hard tissues in the periodontium and, if untreated, can result in tooth loss [3] (Figure 1). Tooth loss, and edentulism in the worst cases, worsens the patient’s quality of life and can lead to impaired chewing function, which carries the risk of malnutrition [4]. In addition, patients may have phonetic, aesthetic, and psychological problems such as loss of self-esteem [5]. Additionally, periodontal disease, as a source of chronic inflammation, has a major impact on the patient’s overall health due to its association with other diseases such as cardiovascular disease [6], diabetes [7], pregnancy and perinatal complications [8], obesity and metabolic syndrome [9], rheumatoid arthritis [10], cancer [11], and Alzheimer’s disease [12]. Unfortunately, patients’ knowledge of periodontal disease is not widespread [13]. The World Health Organization (WHO) points out that oral diseases, including periodontal disease, are an important population problem, having a very high impact on patients’ well-being and generating enormous treatment costs [14]. Given the above, precise, prompt, and predictive diagnosis of periodontal disease is of paramount importance for clinicians. However, despite advances in molecular or microbiological research, the basis of periodontal diagnosis is clinical examination enriched by the evaluation of radiological images.

A characteristic feature of periodontal disease is the formation of periodontal pockets and the loss of the attachment, which can be easily examined using a periodontal probe [15]. Pocket probing is a clinical method of diagnosing the disease and monitoring the progress of treatment. The main measurements are PD (probing depth), GR (gingival recession), and CAL (clinical attachment level). In addition, inflammation, measured by BOP, and tooth mobility are assessed. In radiological image-based diagnostics, attention is paid to the type and extent of alveolar bone destruction. An optimal periodontal examination should include circular probing of each tooth with values recorded at 6 measurement points [16,17]. Periodontal diagnosis is therefore often concentrated at a given point in the periodontium. It should be taken into account that the clinical measurements of PD, GR, and CAL, and the evaluation of radiographs, reflect changes that have already occurred in the periodontium. On their basis, no prognosis of the further course of the disease can be made. With these clinical measurements, we are not able to determine whether they concern active sites with a further tendency to progress or passive sites that may remain stable for many years [18]. A clinical probe examination only allows us to describe the current state of the periodontium. There are no available tests for the clinical prognostication of periodontitis. Therefore, the detailed clinical examination is deepened by the assessment of the BOP index, which is considered an objective indicator of inflammation. The index is assessed as a percentage of bleeding sites in relation to all probing sites [19]. However, the BOP as a stand-alone indicator is not a good predictor of periodontal disease progression because not every site with a positive BOP will experience attachment loss. Achieving a positive BOP in four out of four probings at a given site suggests a 30 percent probability of further attachment loss at that site. However, a negative BOP in four out of four consecutive clinical examinations suggests, with a probability of 98.5%, that no further attachment loss will occur at the point assessed [20]. Patients with deep pockets, advanced adhesion loss, and numerous bleeding periodontal sites after probing are considered to have a higher chance of further attachment destruction than patients with a similar periodontal status but low BOP [21]. However, a mean BOP value that indicates a higher risk of disease progression has not been established [20,22].

**Figure 2:**
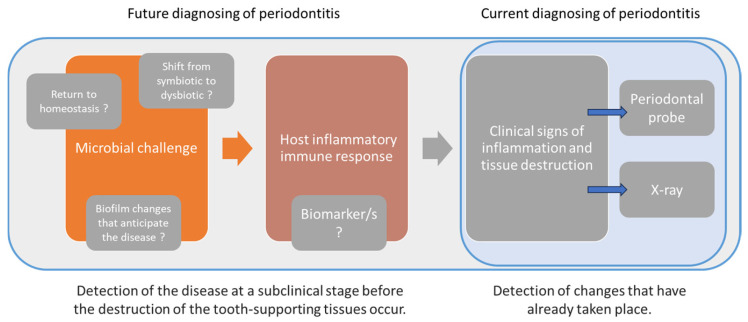
Current and potential diagnostic possibilities on a simplified model of periodontal disease pathogenesis.

Therefore, there is a great need among clinicians to have diagnostic tests that not only describe the periodontal changes that have occurred in the tissues but also allow them to detect disease at a subclinical stage before destruction of the tooth-supporting tissues occurs. This test would enable clinicians to follow the course of the disease and detect areas prone to exacerbation, with the possibility of being used to evaluate the effectiveness of ongoing periodontal therapies (Figure 2). Unfortunately, we do not have such a diagnostic method yet. The hope is molecular diagnostics, which is constantly being developed but not widely used in the clinic. There are numerous studies on biomarkers of periodontal disease. Point-of-care tests are also emerging. However, they have a minor role in the overall diagnostic process.

In this manuscript, we will try to summarize the molecular diagnostic possibilities of periodontal disease and answer the question of why their widespread use is not possible at this stage. We also want to show how the current state of scientific knowledge may influence clinical diagnostics in the future.

## 2. Microbiological Diagnosis

The oral cavity is a complex microbial environment inhabited by more than 700 species of microorganisms [23]. Each person is host to approximately 100–200 species, resulting in a high degree of individual variation. Bacteria responsible for the development of periodontitis form supra- and subgingival biofilms [24]. However, the prevalence of periopathogens in society is greater than the diseases they cause. This indicates a differentiated individual host response to the bacteria, which is responsible for tissue destruction and disease progression [25]. Views on the impact of microorganisms on the periodontium have been changing over the decades and are still evolving. Cessation of oral hygiene leads to plaque accumulation and the development of gingival inflammation. The clinical changes are accompanied by microbiological changes. The early plaque is mainly composed of Gram-positive cocci and bacilli. Over time, it becomes a mature biofilm inhabited by Gram-negative cylindrical bacteria, spirochetes, and rods. The “non-specific plaque” hypothesis assumes that excessive bacterial growth in plaques leads to an increase in its virulence. Therefore, preventive and therapeutic measures should be directed to the removal of plaque formation [26]. The development of the possibility of culturing anaerobic bacteria and their detection by molecular methods have contributed to the “specific plaque” theory, which conjectures that it is the excessive growth of species considered to be periopathogenic that leads to periodontal inflammation, and treatment should be based on targeted elimination of these bacteria [27]. This was confirmed by the groundbreaking work of Socransky and colleagues, who divided plaque microorganisms into five contractual color-coded bacterial complexes, of which the most pathogenic is the red complex consisting of *Porphyromonas gingivalis* (*P.g.*), *Tannerella forsythia* (*T.f.*), and *Treponema denticola* (*T.d.*). It is detected in deep periodontal pockets and associated with active inflammation [28].

*Porphyromonas gingivalis* is considered a key pathogen found in advanced severe periodontitis. The pathogenic potential of this anaerobic bacterium is a result of the production of many virulence factors, such as peptidylarginine deiminase (PPAD) and the lipoprotein RagB, a component of the nutrient acquisition system and gingipains [29]. It also produces adhesins that allow it to bind to epithelial surface, erythrocytes, and other oral structures. The number of proteinases it produces causes the breakdown of tissue integrity [30]. *Treponema denticola* and *Tannerella forsythia* are also Gram-negative anaerobes with similar characteristics but lower virulence [31].

*Aggregatibacter actinomycetemcomitans* is a Gram-negative bacterium associated not only with advanced periodontal disease in adults but also with periodontitis occurring in young people and with other severe infections outside the oral cavity [32]. It is known that the above microorganism releases an endotoxin that induces severe inflammation in the periodontium (lipopolysaccharide, LPS). However, it has been demonstrated that in addition to LPS, this bacterium secretes two specific exotoxins—leukotoxin A and cytolethal distending toxin (CDT) [33].

Further studies by Socransky confirmed that periodontitis is not a classical infection and that the bacteria causing it do not fulfill Koch’s postulates [34]. This gave rise to the “ecological plaque” hypothesis. This theory assumes that in a healthy state, there is a balance between the host and its microbiome. Changes in the microenvironment lead to excessive growth of certain species, which begin to act as opportunistic pathogens that initiate inflammation. The microbial imbalance is dysbiosis. Therefore, the term “multi-microbial synergy and dysbiosis” is used to refer to this phenomenon [35]. In a bacterial plaque environment, different bacteria have distinct functions and collectively form a microflora that can lead to the development of inflammation. As a result of inflammation, tissue breakdown occurs, which is a source of nutrients for pathogens. Further growth of these pathogens exacerbates the inflammation. This leads to a vicious circle where dysbiosis fuels inflammation and inflammation intensifies dysbiosis. This sustained inflammation is not self-limiting [36]. In view of the repeatedly confirmed role of the bacterium *P. gingivalis*, which has the ability to transform symbiotic microflora into dysbiotic one—due to its numerous virulence factors, the ‘key pathogen theory’ has emerged. This hypothesis, in contrast to the “specific plaque” hypothesis, assumes that even a small number of *P. gingivalis* cells can have a tremendous impact on the subgingival biofilm [36]. In 2020, a new theory of the etiology of periodontitis emerged called the IMPEDE (Inflammation-Mediated Polymicrobial Exacerbation) model. This model assumes that it is inflammation that leads to dysbiosis, which drives the transition of the oral health condition to periodontitis. The following is still relevant; therefore, the question of whether the dysbiotic changes in the biofilm are the cause of the disease, or whether it is the inflammation that leads to a change in the conditions of the subgingival environment that results in dysbiosis, is still open [37]. There has also been a recent hypothesis that subgingival plaque mimics human tissue in both structure and function. Thus, inflammation results from the disruption of the normal function of a healthy biofilm, which begins to stimulate the host’s defense mechanisms, leading to excessive inflammation that is difficult to regulate [38].

**Figure 3:**
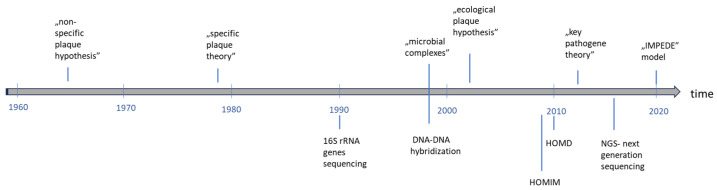
Hypotheses on the role of bacteria in the etiopathogenesis of periodontal disease and milestones in the study of the oral microbiome on a timeline. (HOMIM—Human Oral Microbe Identification Microarray, HOMD—the Human Oral Microbiome Database, IMPEDE—Inflammation-Mediated Polymicrobial Emergence and Dysbiotic Exacerbation).

Due to the strong role of bacterial factors in the etiology of periodontitis, relationships between the presence and quantity of specific microorganisms in pockets have been studied for use in diagnostic and therapeutic procedures [39]. Since subgingival plaque is a biofilm, the object of microbiological examination should not be individual cells, but rather the entire biofilm structure, where the presence of some species is determined by the colonization of others [40]. Most of the bacterial species that populate the oral microbiome cannot be cultured under laboratory conditions, and previous methods such as bright-field and dark-field microscopy are of little use in these cases [41]. New molecular methods for identifying bacterial species include hybridization and polymerase chain reaction (PCR). The DNA–DNA checkerboard Socransky’s method proved to be a breakthrough. Methodologically, it relied on the use of whole-genomic probes, which were a purified collection of DNA extracts from pure cultures. DNA isolated from bacterial samples was attached to the surface of a nylon or nitrocellulose filter. Then, the filter was subjected to hybridization with DNA probes from at least 40 bacterial species. The number of individual species was determined by the intensity of the fluorescent light from the molecular probes. Based on the frequency of detection of subgingival species, five color-coded complexes were proposed [28,42]. Almost simultaneously, in the 1990s, the 16S rRNA gene was discovered. It is a component of the 30S subunit in the prokaryotic ribosome. All bacteria have it in their genome and there are unique sequence differences in it that allow the microorganism to be classified to a particular genus or even species [43]. With the advent of modern diagnostic techniques, polymerase chain reaction (PCR) began to be used, allowing cloning and sequencing of bacterial genomes with their subsequent analysis. The standard PCR method is a qualitative method [44]. In addition to the presence of bacteria, the development of quantitative polymerase chain reaction (qPCR) allows the quantification of the microbial composition of the sample [45,46]. Other modifications of the PCR technique have also found applications in diagnostics of periodontitis. Nested PCR is used when there is a need to increase the accuracy or specificity of the test. It involves the sequential use of two sets of primers; the first is used to amplify the target sequence and the resulting amplicon is then used as the sequence for a second amplification [47,48]. When a polymicrobial sample is analyzed, such as a dental biofilm, a multiplex polymerase chain reaction can be used. This makes it possible to identify organisms or genes using a single reaction and minimizes the required sample volume [49]. In 2009, a microarray for the identification of microbes in the human mouth (HOMIM—Human Oral Microbe Identification Microarray) was used. This method allowed the detection of 300 species across different periodontal conditions [50]. Thanks to this, a dedicated repository has been created—the Human Oral Microbiome Database (HOMD) [51]. The use of small probes allowed the identification of nearly 300 bacterial phylotypes in a single sample [52]. Finally, in 2016, the HOMINGS system was developed using next-generation sequencing (NGS). This allowed the identification of approximately 700 phylotypes in a single sample [53] (Figure 3). Due to the complex structure of the subgingival biofilm, species identification alone does not describe the relationship between the bacteria forming the microbiome. Bacteria have a wide range of possibilities for interaction and adaptation through modulation of protein synthesis, metabolism, and the secretion of small molecules. A comprehensive understanding of the functional diversity of the subgingival microbiome requires the combination of information based on DNA and RNA with bacterial products and proteins, namely through metabolomics and metaproteomics [54].

Studies of the subgingival microbiome conducted by sequencing the gene encoding 16S rRNA have shown that *Actinomyces naeslundii*, *Rothia dentocariosa* and *aeria*, and *Streptococcus sanguis* are detected in areas with healthy periodontium. In sites with periodontitis, abundant growth was found for *Porphyromonas gingivalis* and *endodontalis*, *Prevotella intermedia*, *Tannerella forsythia*, *Treponema* (*denticola socranskii*, *maltophilum*, and *lecitinolythicum*), *Selenomonas sputigena*, *Parvimonas micra*, *Peptostreptococcus saphenum*, and *Fretibacterium fastidiosum* [55]. In addition, core bacterial species such as *Fusobacterium nucleatum* play an important role in the transition from health to disease, possibly serving as a metabolic anchor for other community members. *F. nucleatum* can be a prerequisite for late colonizing anaerobic species [56].

Advances in molecular diagnostics made it less expensive and resulted in a range of commercially available tests that allow the molecular evaluation of selected bacterial species from periodontal pockets. The results can report the bacterial count in the sample or the content of specific strains of pathogens. Examples of available microbiological tests are PET (MIP Pharma holding GmbH, Blieskastel, Germany), enabling quantitative evaluation of up to nine periopathogens per sample by real-timePCR; IAI PadoTest (ParoX GmbH, Leipzig, Germany) for the detection of key pathogens (*P.g*, *T.f*, *T.d*, *Prevotella intermedia* (*P.i*), and *Filifactor alocis*) by multiplex rtPCR; PerioPOC (GenSpeed Biotech GmbH, Gebaude B, Austria), a semi-quantitative method with a detection limit of 10^4^ CFU/l (detects *P.g*, *T.d*, *T.f*, *P.i*, and *A.a*); and MyPerioPath (OralDNALabs, Flying Cloud Eden Prairie, MN, USA), which classifies pathogens into three risk groups on the basis of PCR testing. The Test PadoBiom Kit (ParoX GmbH, Leipzig, Germany) uses next-generation sequencing (NGS) of GCF samples, provides the ability to assess bacterial diversity and pathogenicity, and detects resistance genes to antibiotics such as beta-lactams, quinolones, nitro-imidazole derivatives, tetracyclines, and macrolides.

Despite studies confirming the validity of the use of microbiological tests to detect putative periodontopathogens, this topic needs more extensive studies. Such tests give us information about the possible number and types of bacteria present in periodontal pockets, which does not significantly affect clinical management. Laboratory tests make sense when the information they provide helps to plan treatment and apply optimal therapy. Many infectious diseases are treated without a thorough microbiological diagnosis because the probability of detecting a known pathogen for a given infection is very high and experience shows that standard anti-infective therapy is effective. To diagnose gingivitis or periodontitis, a microbiological diagnosis is not necessary because clinical examination is sufficient. With the modern approach to the etiology of periodontitis, diagnosing single bacterial species for the clinician becomes meaningless, because the detection of the presence of potentially pathogenic microorganisms is not necessarily indicative of disease, nor does it provide information about its activity or propensity for progression. Despite the possibility of using sophisticated laboratory methods to detect bacteria both quantitatively and qualitatively, this knowledge does not translate into a change in the therapeutic process. It is important to note that current guidelines for antibiotic prescriptions in periodontology are not based on the detection of specific pathogens but on a clinical assessment of the severity of the disease [57,58]. However, an accurate microbiological diagnosis may be useful for treatment planning for patients who need to eradicate bacteria with exogenous pathogen characteristics (i.e., *A.a.* and *P.g*), particularly in patients classified before 2018 as having aggressive periodontitis and in patients with refractory forms of periodontitis. Taking into account the above, precise microbial testing could help to select subjects who benefit from antibiotic therapy, assist in selecting the right antibiotics, and contribute to minimizing the overuse of antibiotics in society [59]. Such diagnostics do not require species assessment but only the identification of antibiotic resistance genes. Knowledge of resistance to antibiotics of a certain type would reduce the number of unsuccessful antibiotic treatments.

Another desirable application of microbiological diagnostics can be the detection of changes in the biofilm that anticipate the onset of disease. A change in the microbiome always precedes an inflammatory response, so early detection of changes is desirable and may allow identification of patients who are ‘at risk’ before clinical signs of inflammation in the periodontium occur [60]. A shift in the microbiome from symbiotic to dysbiotic can alert clinicians to the likely onset of disease. The same applies to patient monitoring during maintenance therapy visits. The impact of the bacterial agent on the recurrence of active inflammation is undisputed. Early detection of changes in the microbiota would make it possible to individually determine when the next prophylactic intervention is indicated. However, the detection of certain pathogens has proved less useful than an examination of the overall bacterial load. In addition, it was proven that maintenance visits set every three months provide stability for almost all patients and those carried out at intervals longer than six months increase the risk of relapse, so a microbiological evaluation is not strictly necessary [61].

The microbiological tests may also be a measure of the return to homeostasis between host and plaque bacteria after effective periodontal treatment. Chen et al. developed the subgingival microbial dysbiosis index (SMDI) to be able to describe changes in the microbiome [62]. The SMDI is based on 49 discriminating species. Among them, the top species associated with periodontitis were *Treponema denticola*, *Mogibacterium timidum*, *Fretibacterium* spp., and *Tannerella forsythia.* In contrast, *Actinomyces naeslundii* and *Streptococcus sanguis* were the top health-associated species. Finally, *Treponema*, *Fretibacterium*, and *Actinomyces* are used to calculate the simplified SMDI [62]. The SMDI was used to compare the microbiome before and after scaling and root planing. There was a significant decrease in the SMDI between day one after therapy and stabilization up to three months. These changes indicated a decrease in dysbiosis after nonsurgical periodontal treatment [63].

## 3. Molecular Diagnostics of Gingival Crevicular Fluid (GCF) and Saliva

The effect of microorganisms on periodontal tissues is bidirectional. One direction is direct action, referring to the release of enzymes that destroy host structures (proteases, collagenases, etc.). The other is an indirect impact via bacterial cytotoxins and involves the stimulation of destructive actions of the patient’s own immune system. In response to the detection of substances of bacterial origin, polymorphonuclear neutrophils (PMNs) initiate the production of pro-inflammatory factors. MMPs are responsible for the destruction of connective tissue, and PGE2 (prostaglandin E2) stimulates osteoclasts to destroy the bone structure [64] (Figure 1). In this complex immunological reaction, many pro-inflammatory biomolecules are secreted. They may originate from the host or are the products of tissue destruction [65]. Many of them are studied as possible periodontitis markers. Biomarkers are searched for in saliva, subgingival plaque, tissue biopsies, and gingival crevicular fluid (GCF).

The gingival crevicular fluid (GCF) itself is a plasma filtrate and/or inflammatory exudate and can be collected from the gingival crevice/periodontal pocket. The strong vascularity of the periodontal tissues contributes to the continuous filtering of fluid into the gingival crevice. The components of the fluid originate from both the host and subgingival microorganisms. Among the important host-derived components are inflammatory markers including enzymes, cytokines, and interleukins [66,67]. The association of increased GCF volume with increased severity of periodontal inflammation is well-documented [68,69,70]. It has been suggested that increased GCF volume and the appearance of bleeding after probing is one of the earliest signs of disease and the increase in GCF volume itself may be a sign of subclinical ongoing inflammation [71]. GCF collection is non-invasive and provides information about a specific site in the periodontium. Although reviews [67,72] have suggested that there is considerable potential for the future application of GCF-based tests, the question is whether this will lead to improved disease detection and patient outcomes. At present, there is a large gap between diagnostic capability and clinical application. A positive test result should guide the clinician to change the treatment plan accordingly; however, at the moment, it only provides more information about the course of the disease. Additionally, there are no dedicated laboratories for analyzing GCF components, and chair-side testing is not available for most markers. Therefore, more research is needed to identify reliable biomarkers for periodontal disease monitoring. Such biomarkers for periodontal diagnostics need to be optimized through studies combining biochemical and clinical periodontal data. Validation of candidate biomarkers in large populations is also required. Taking into account its unparalleled affinity for periodontal tissues, GCF should be the medium of choice. It is excellent for site-specific diagnosis.

However, saliva collection provides information from the entire oral cavity, not only from specific sites of periodontium. Saliva comprises secretions from salivary glands, oral mucosa cells, blood, and GCF. Saliva as a medium is therefore more practical, easier, and cheaper to collect. It contains discharges from parotid, submandibular, sublingual, and many small salivary glands, the secretion of which is influenced by many environmental and psychological factors. In addition, saliva contains mucins and cellular debris. Similarly to serum, it contains DNA, mRNA, microRNA, proteins, metabolites, and microbiota [73]. The variety of molecules makes it a difficult medium to work with. Therefore, in the case of periodontitis, it is still secondary to GCF [74]. Saliva can be collected stimulated, unstimulated, and as a swab from the floor of the mouth or gums, in which case it contains more GCF [75]. Saliva is a convenient medium to collect and its composition is very rich. Proteotome saliva analysis has revealed the presence of 3000 different biomolecules [76]. There are about 3500 publications on salivary biomarkers [75]. However, saliva testing is not yet a routine component of dentistry. However, attempts are being made to use saliva as a medium for the diagnosis of many general diseases, particularly in oncological diagnosis and the detection of autoimmune diseases, systemic microbial infections, or diabetes [77]. Capillary electrophoresis mass spectrometry-based saliva metabolomics can distinguish pancreatic cancer from oral cancer, breast cancer, and cancer-free controls [78]. Biomarkers for lung cancer can improve early detection beyond the use of computed tomography scans [79], as well as detect breast cancer [80,81]. Identification of reliable saliva biomarkers can provide a convenient non-invasive way for cancer detection [82]. Saliva is therefore a medium with a very high diagnostic potential, but its use in the diagnosis of periodontal disease by dentists is practically non-existent.

There are numerous research studies on the role of inflammatory mediators, host-derived enzymes, oxidative stress markers, and tissue breakdown products in oral fluids. The most extensively studied molecules are IL-1β, IL-6, IL-8, MMP-8, TNF-α, and PGE2 [83,84,85,86,87]. Meta-analysis performed for salivary biomarkers showed the highest sensitivity for the diagnosis of periodontitis for IL-1β (77.8%) and MMP-8 (72.5%), and the highest specificity for MMP-9 (81.5%) and IL-1β (78%) [86]. For GCF molecules, the highest sensitivity (76.7%) and specificity (92%) were shown for MMP-8 [85]. Bone turnover markers are also of interest. Osteoclastic activity is mainly regulated by receptor activator of nuclear *κ*B (RANK), its ligand (RANKL), and OPG (osteoprotegrin). The RANKL/OPG ratio is important in determining bone resorption. Increased levels of RANKL and decreased levels of OPG were noted in periodontal inflammation [88]. The RANKL/OPG ratio may be a predictor of sites at risk of progression.

Despite many years of searching, no single biomarker of periodontitis has been identified that, when assayed from saliva or GCF, would provide information about what has happened, is currently happening, and will happen in the periodontium. That is why the idea of combining several biomarkers emerged. It was shown that combining biomarkers shows better diagnostic accuracy in the detection of periodontal disease and potentially provides information on the stages of disease [89,90]. The most promising combinations comprise salivary molecules and the presence of known periopathogens. High salivary concentrations of MMP-8, IL-1β, and *P. gingivalis* were associated with deep pockets and alveolar bone loss, and high levels of MMP-8 and IL-1β with bleeding on probing. The CRS (cumulative risk score) is a mathematical model used to define the individual assessment of the risk of developing periodontal disease [91]. Three selected salivary biomarkers represent three components of periodontal inflammation: periopathogens (*P. gingivalis*), cytokine production (IL-1β), and tissue degradation (MMP-8). The CRS index had a strong association with moderate to severe periodontitis [92], similar to the determination of the presence of *P. gingivalis* with IL-1β and PGE2 levels [93]. The 2023 systematic review of the accuracy of multiple molecular biomarkers in oral fluids revealed two biomarker combinations with high diagnostic accuracies. In saliva, combinations of IL-1β, IL-6, and MMP-8 have superior properties for the detection of periodontitis [94].

Matrix metalloproteinases (MMPs) are zinc-dependent endopeptidases. These enzymes have the ability to degrade basement membranes and the extracellular matrix, which enables tissue remodeling and cell movement during physiological processes and inflammatory states in the body. MMPs are secreted by many host cells such as polymorphonuclear leukocytes, macrophages, fibroblasts, bone cells, epithelial cells, and endothelial cells [95]. The main MMPs derived from neutrophils are MMP-8 (collagenase-2) and MMP-9 (gelatinase B). MMP-8 is produced during the maturation of PMN cells and then stored in their granules. During the course of inflammation, the content of the granules is released into both saliva and the GCF. MMP-8 is responsible for 90–95% of collagenolytic enzyme activity in the GCF. Its levels are much higher in patients with periodontal disease than in healthy patients. MMP-8 and MMP-9, as the main collagenolytic enzymes in saliva and gingival crevicular fluid, are responsible for collagen degradation in gingivitis and periodontitis [96,97,98]. That is why MMPs were analyzed as diagnostic and prognostic markers. Examination of MMP-8 and MMP-9 concentrations in saliva, together with confirmation of the presence of the bacterial red complex and analysis of orthopantomograms, had predictive value [99]. High concentrations of MMP-9 and MMP-13 showed active sites with disease progression [100]. Long-term high levels of MMP-8 in the GCF indicated a high risk and poor responses to therapy [101]. However, most analyses did not take into account the fact that the amount of MMPs corresponds to the presence of the cells through which they are secreted, rather than to direct tissue destruction [102].

Nevertheless, MMP-8 is still the most promising biomarker for periodontal disease, with high specificity and sensitivity. These findings were used to develop a point-of-care test (PerioSafe^®^,Dentognostics GmbH, Solingen, Germany) to measure active MMP-8 in saliva as an adjunct to clinical diagnostics. The procedure itself involves taking a sample at least 30 min after ingestion of the last meal or drink [101]. Although this test has a low sensitivity (33%), it has a high specificity of up to 93% for detecting periodontitis. When combined with age and smoking, sensitivity was improved to 82.5% and specificity was increased to 84.4%. This test may be useful in periodontal screening in conjunction with patient characteristics [103]. There are attempts to use other chair-side tests such as a lateral flow metalloproteinase 9 point-of-care test (MMP-9 LFT POC test). Its diagnostic ability to detect periodontitis was appropriate, with a sensitivity of 0.92 and a specificity of 0.72 [104].

Many opportunities are opening for clinicians with the introduction of chair-side point-of-care tests (POCTs). These involve taking a saliva or GCF sample from the patient, inserting it onto a special microchip, and reading the result a few moments later, while the patient is still in the chair. The test result tells us about the presence of a specific biomarker at the site from which the sample was taken and could be used to screen patients for periodontal disease. Such tests could be applied in many places providing health services to the public, i.e., general medical and dental practice offices, nursing homes, outpatient clinics, and potentially for self-testing at home [105]. Chair-side diagnostics provide instant results, thereby reducing time and costs compared to laboratory diagnostics. However, analysis of the subgingival microbiome is not routinely used in periodontal clinics.

## 4. Gene Variation in Periodontitis and Its Impact on Diagnostic and Therapeutic Management

Periodontitis is a complex, multifactorially inherited disease, dependent on multiple alleles, and its phenotypic picture depends on the interaction between genetic predisposition and environmental factors [106]. As with other complex diseases, it is slowly progressive, presents a relatively mild phenotype, and is chronic in nature [107]. Periodontitis is polygenic and each gene has low penetration; therefore, it can be called a disease-modifying gene. It is estimated that 10 to 20 disease-modifying genes are involved in the disease [108]. Gene polymorphisms can cause changes in protein coding or expression, which may result in changes in innate or acquired immunity and therefore may determine disease or protect against it. A single nucleotide polymorphism (SNP) is an important genetic marker, along with copy number variation. Nowadays, SNPs can be detected in large amounts using high-throughput techniques [108]. Many studies were performed to understand gene polymorphisms in the etiology of periodontitis. The most widely studied were genes *IL-1A* (interleukin-1A), *IL-1B* (interleukin-1B), *IL-4* (interleukin-4), *IL-6* (interleukin-6), *IL-10* (interleukin-10), *TNFα* (tumor necrosis factor α), *FcγR* (the Fcγ receptor), *VDR* (vitamin D receptor), *CD14*, *TLR2* (toll-like receptor 2), *TLR4* (toll-like receptor 4), and *MMP-1* (metalloproteinase-1) [108,109,110,111,112]. However, the frequency distribution of the polymorphisms of the indicated genes does not correlate with the prevalence of periodontitis. It is likely that a single gene may have little influence on disease initiation and pathogenesis and other genes may interact. At the same time, no single gene has a dominant influence. The role of genetics in the pathogenesis of periodontitis is difficult to explain, as there are complex interactions between genes and their polymorphisms and between genes and environmental factors. Therefore, polygenic models seem to be more accurate than monogenetic models, but the progress of the latter is very slow [113]. Additionally, the distribution of polymorphisms varies between ethnic groups, and differences due to ethnicity are often found in periodontitis [113].

The *IL-1* gene cluster was among the first to be analyzed, and SNPs in the *IL-1* gene cluster were proposed for use in genotyping periodontal patients [114,115]. After the study by Kornman, which found an association between polymorphisms in the *IL-1* gene cluster and the severity of periodontitis in non-smokers, genetic studies in periodontology began [116]. There are three genes that regulate the production of interleukin-1: *IL-1A*, *IL-1B*, and *IL-1RN* (interleukin receptor antagonist). These genes are located on chromosome 2. The first two control the production of pro-inflammatory IL-1α and IL-1β. *IL1-RN* codes for the synthesis of IL-ra, which inhibits the secretion of IL-1α and IL-1β. In Caucasians, the polymorphisms *IL-1A* −889T/C and *IL-1B* 3953/4 C/T are associated with chronic periodontitis [117]. On this basis, a commercially available genetic susceptibility test was developed (Periodontal Susceptibility Test, Straumann, Waltham, MA, USA) to determine the risk of severe chronic periodontitis. The test detected the simultaneous presence of *IL-1RA* + 4845 (*IL-1RA* + 4845 is being used because it is easier to identify than the *IL-1A* −889 polymorphism and it is concordant with it) and *IL-1B* + 3954. The presence of both polymorphisms meant that the patient was considered “genotype-positive” and predisposed to increased IL-1β release [118]. Genotype-positive, nonsmoking patients were found to be 6.8 times more likely to have severe chronic periodontitis than genotype-negative patients [117]. However, analysis of the clinical trials utilizing this genetic test has yielded inconclusive results, as did the attempt to link the result of a genetic test to bleeding on probing, loss of clinical attachment, and loss of bone or teeth [118]. Therefore, the genetic test for periodontitis resulted in unclear benefits and subsequent studies were unable to confirm the practical utility of the observed associations [119].

At the individual level, the expression of the disease depends on genetic, bacterial, and environmental factors. The link between them is epigenetics. Epigenetics is the science that deals with heritable mechanisms of gene expression that are not dependent on changes in the DNA sequence. Consequently, epigenetic mechanisms regulate gene expression and allow cells with identical genetic material to perform a variety of functions in the body. Environmental factors such as microorganisms or smoking may affect gene activation and cell phenotype [120].

Genetic factors are not the only risk factors for periodontitis. Genetic predisposition means that a patient has an inherited susceptibility to develop the disease, but it does not mean that a person with such a genetic tendency is doomed to its development. The validity of commercially available genetic tests for complex diseases is therefore questioned, comparing their accuracy to that of horoscopes [121]. That is why genetic profile testing for patients is not used in clinical practice. The tests are expensive and do not add diagnostic value. In addition, no protocols have been developed for following up on the identification of a specific gene variant in a patient. In periodontology, it is questionable if every patient should receive different, personalized treatment according to their genetics. There were no analyses to determine whether genetic screening in patients with or without periodontitis is economically and ethically justifiable. However, there is space for future use of human genomics in the characterization of disease subtypes and personalized care plans. In particular, cases of children and young adults with periodontitis would benefit from such diagnosis. Familial aggregation of periodontitis in the aforementioned patients was confirmed in single-family and large twin studies [122,123]. However, at the moment, it is clear that single-gene inheritance is rare and is mainly considered in the case of genetic syndromes [124]. There are many putative loci and SNPs that may confer a genetic risk for periodontitis but they lack universal validation across different settings and populations [125]. A validated polygenic risk score (PRS) that aggregates the information from hundreds of thousands of genetic loci and polymorphisms measured via genome-wide association studies (GWAS) is needed to identify healthy people with susceptibility to the disease. Unfortunately, such a PRS has a strong risk of bias that is connected with ethnicity and ancestry. Genetic association signals discovered in GWAS studies in populations of European descent are not always transferable to non-European populations [126]. In order to generalize, the PRS needs to be verified in populations from different ethnic backgrounds.

In summary, there is huge potential in such studies; however, time is needed for further research into the genetic links of periodontitis so that this knowledge can be applied routinely in practice. Before this, new approaches will be supported by high-quality evidence, and information about the patient’s family history of periodontitis can still be used to identify susceptible subjects with an elevated risk of periodontitis [127].

## 5. The Role of Molecular Diagnosis in Classifying Periodontal Disease

The current classification of periodontal diseases was established in 2017 [128] and replaced the 1999 classification developed by the International Workshop for Classification of Periodontal Diseases and Conditions [129]. It covers the three main forms of periodontitis, i.e., necrotizing periodontal disease, periodontitis as a symptom of systemic disease, and periodontitis (previously divided into chronic and aggressive). The progression of the disease and further risk of progression are described by stages and grades, which may be modified as new scientific evidence emerges. Stages describe the progression of the disease and the complexity of the treatment process. Grades provide additional information about the progression of the disease and its biological features [130]. The previous classification divided periodontitis into chronic (CP) and aggressive (AP) forms. To make a diagnosis of chronic periodontitis, an aggressive form of the disease had to be excluded. Clinically, CP was more common in adults (but could also be diagnosed in children and adolescents), tissue destruction went hand in hand with plaque accumulation, and subgingival calculus deposits were often found. In addition, the course of the disease was slow or moderate with possible episodes of rapid tissue destruction [131]. In contrast, the primary features of AP were rapid attachment, bone loss, and a family history of the disease. The secondary clinical features were an imbalance between the amount of dental deposits and periodontal tissue destruction, elevated proportions of *Aggregatibacter actinomycetemcomitans* (*A.a*) or *Porphyromonas gingivalis* (*P.g*), phagocyte abnormalities, and macrophages hyperresponsiveness [132]. Clinical diagnostic assumptions can be considered subjective and in many published papers, the diagnosis of AP has been considered incorrect or at least questionable [133], as confirming secondary molecular features was not mandatory. Microbiological diagnostics have been attempted to distinguish between AP and CP on the assumption that the detection of *Aggregatibacter actinomycetemcomitans* (*A.a*) would confirm the diagnosis of AP. In the late 1970s, *A.a*. was isolated from deep pockets of patients with localized aggressive periodontitis (LAP) [134], which was later confirmed by the finding that the prevalence of this bacterium in LAP was very high [135,136]. Subsequent work confirmed that 96.5% of LAP patients carried *A.a.*, while only 20.8% of CP patients and 16.9% of periodontally healthy patients harbored this bacterium [137]. However, the results of other studies were inconclusive. When AP and CP patients were tested for *A.a.* and *P.g.*, *Aggregatibacter actinomycetemcomitans* was detected in 54% of AP patients and 47% of CP patients. Inversely, *Porphyromonas gingivalis* was detected in 67% of CP patients and 52% of AP patients [138]. In addition, only the JP2 A.a. strain was found to have the typical characteristics of an exogenous pathogen and the others showed characteristics of opportunistic bacteria [139]. The JP2 clone strains are highly prevalent in human populations living in northern and western parts of Africa and in populations originating from these geographical regions. Only sporadic signs of dissemination of the JP2 clone strains to non-African populations have been found. *A. actinomycetemcomitans* is a source of leukotoxin (LtxA) and cytolethal distending toxin (Cdt). LtxA is able to kill human immune cells and its production is enhanced in JP2 clones. This is why the highly leukotoxic *A. actinomycetemcomitans* JP2 clone is associated with rapidly progressing periodontal disease and is frequently detected in LAP (i.e., Localized Stage III Grade C Periodontitis). Patients colonized with the JP2 strain can transmit it to family members and partners, making its eradication difficult [140,141].

It is also hypothesized that LAP may develop into GAP over time, which simultaneously leads to the development of more complex microbiota beginning to resemble those in CP [123]. The mere detection of *A.a.* in the patient’s plaque sample could therefore not confirm the diagnosis of AP, so it remained an ancillary test. PCR-based diagnostic tests, although becoming cheaper and more widespread, have not become an essential diagnostic tool. They are now readily available and allow confirmation of a certain number of pathogens, including *A.a.* and *P.g.* Early detection of the mentioned pathogens could prevent rapid periodontal attachment loss. The virulence factors of *A.a.* (LtxA) and *P.g*. (gingipains) are known and new virulence blocking strategies are emerging. Anti-virulence therapy fights periodontal pathogens by neutralizing their virulence properties and may be an alternative to antibiotic treatment. Host immune modulation by phytocompounds and oral microbiota replacement are new options for periodontitis treatment and prevention [142].

Due to the fact that periodontitis is a multifactorial disease and its clinical expression depends on the imbalance between risk factors (including dental plaque bacteria) and the host response, immune disorders may be crucial. People with impaired neutrophil function, e.g., leukocyte adhesion deficit, Kostmann syndrome, Chediak–Higashi syndrome, Papillon–Lefevre syndrome, or Down syndrome, are predisposed to severe periodontitis [143]. It was found that people with periodontitis at a young age may have reduced neutrophil chemotaxis and phagocytosis [144,145]. Their hyperactive neutrophils secrete excessive amounts of oxygen radicals [146]. However, a recent systematic review assessing the GCF composition in patients with chronic and aggressive periodontitis did not show sufficient evidence for differences between these diseases [147]. Complicated and expensive methods for determining cytokines from GCF are completely unavailable to clinicians. The use of the term aggressive periodontitis was therefore common without a proper methodology for diagnosing the disease. The current 2018 classification does not distinguish aggressive periodontitis [128]. It was stated that differences in etiology and pathophysiology are required to indicate separate periodontal diseases and clinical manifestations, and severity is not enough to support the concept of different diseases. However, it remains undisputed that significant differences in the clinical presentation of the disease are present, suggesting population variations in susceptibility and/or exposure. Even localized periodontitis diagnosed in young people cannot be confirmed by defined etiological or pathological elements and the disease mechanisms are poorly understood [128].

The new classification is still open and allows for the inclusion of biomarkers in a case definition system, particularly in patients who are more likely to develop progressive severe generalized periodontitis, are less responsive to standard plaque control methods, and theoretically may have periodontitis severely affecting general disease. In these patients, standard clinical diagnostics may be insufficient and biomarkers that are currently available may be useful. Biomarkers may increase diagnostic accuracy and allow better assessment of severity. Therefore, the proposed classification framework allows the introduction of validated biomarkers into the case definition system. Specific biomarkers and their thresholds may be incorporated into the system as evidence becomes available [148]. Despite the possibility of inclusion of widely understood periodontal disease biomarkers present in the GCF, saliva, or serum during staging and grading, the classification does not, at present, indicate specific molecules or the extent of their diagnostic value. The question of molecular diagnostics remains open and its widespread use is not yet apparent.

## 6. Summary

Microbiological, molecular, or genetic diagnostics in periodontal disease provide scientists with a wealth of knowledge about the processes that occur in the healthy and inflamed periodontium. However, the practical use of these components that make up the picture of periodontitis currently seems impossible. Even state-of-the-art diagnostic methods that allow us to learn about the metabolome and proteome of oral fluids only allow us to assess the presence of certain molecules rather than their joint action. The complex interactions in the microbiome, the intersection of many metabolic pathways, or the interplay of genes or genes with the environment make periodontal disease diagnosis similar to a 3D jigsaw puzzle, whose components interact with each other and are not constant.

When considering the complex etiology of periodontitis, it is unlikely that a single lab test will address all issues. The perfect periodontal diagnosis of the future should combine clinical, radiological, and laboratory examinations. Thus, to date, no specific protocols have been developed in which molecular tests would have a significant impact on increasing the diagnostic and therapeutic quality of the management of patients with periodontal disease. However, the continuing development of new diagnostic methods and the detailing of the knowledge of immune reactions in the periodontium will certainly be a step forward in the possibilities of periodontal diagnostics in the future. Valuable solutions will be transferred from the laboratory to clinical practice. Until this happens, clinical examination together with radiological assessment remains the primary means of periodontal evaluation and the patient’s diagnosis and treatment are based on it.

## Data Availability

Not applicable.
